# Oesophageal neuroendocrine tumours—case series of a rare malignancy

**DOI:** 10.1093/jscr/rjac582

**Published:** 2022-12-20

**Authors:** Amanda L Nikolic, James Gullifer, Mary Ann Johnson, Michael W Hii

**Affiliations:** Department of Hepatobiliary and Upper Gastrointestinal Surgery, St Vincent’s Hospital Melbourne, Fitzroy 3065, Australia; The University of Melbourne, Department of Surgery, St Vincent’s Hospital Melbourne, Fitzroy 3065, Australia; Department of Anatomical Pathology, St Vincent’s Hospital Melbourne, Fitzroy 3065, Australia; Department of Hepatobiliary and Upper Gastrointestinal Surgery, St Vincent’s Hospital Melbourne, Fitzroy 3065, Australia; Department of Hepatobiliary and Upper Gastrointestinal Surgery, St Vincent’s Hospital Melbourne, Fitzroy 3065, Australia; The University of Melbourne, Department of Surgery, St Vincent’s Hospital Melbourne, Fitzroy 3065, Australia

**Keywords:** case series, neuroendocrine neoplasm, oesophageal neoplasm

## Abstract

Oesophageal neuroendocrine neoplasms (NENs) are rare tumours. Neuroendocrine carcinomas (NECs) are the highest grade of NENs, with aggressive biological behaviour and poor outcomes. No standardized treatment pathways exist for these tumours, with management being individualized based on patient and tumour factors. We present five cases, four men and one women between 63 and 68 years old, who were diagnosed with symptomatic primary oesophageal NECs. Three were diagnosed with localized disease, and two were diagnosed with metastatic disease. Endoscopy, biopsy and staging scan results are outlined. Two patients received neoadjuvant chemotherapy. Three patients with localized disease underwent oesophagectomy. Two of these patients received neoadjuvant chemotherapy. Four patients have succumbed to their disease, with a median survival following a diagnosis of 18 months (5–34 months). This case series highlights the variability of presentation and stage at diagnosis of oesophageal NECs. Multimodal treatment is commonly utilized; however, outcomes are universally poor. Further research is required to determine the optimal treatment regimen for oesophageal NENS.

## INTRODUCTION

**Figure 1:**
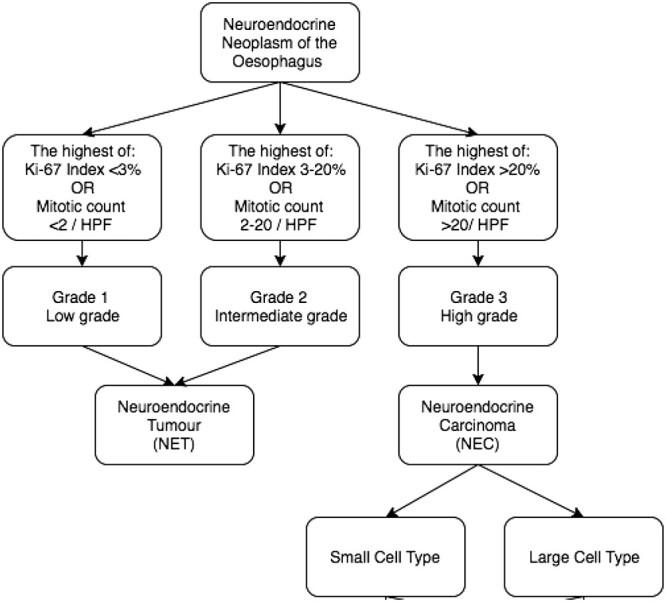
2010 WHO classification system for oesophageal neuroendocrine neoplasms [5].

Oesophageal neuroendocrine neoplasms (NENs) encompass a group of rare and heterogeneous tumours that are associated with very poor outcomes [1]. They constitute only 2% of malignant tumours of the gastrointestinal tract, with the majority located in the small bowel [2]. The classification of NENs was changed by the World Health Organisation in 2010 and updated in 2019 [3–5]. The new classification reflects the clinical and prognostic course of these tumours and grades them based on their mitotic count and Ki-67 index [3–5] (Fig. 1). Grade 1 and 2 tumours, labelled neuroendocrine tumours (NETs), behave in a relatively indolent fashion. Grade 3 tumours, known as neuroendocrine carcinomas (NECs), behave aggressively and have a poor prognosis [6]. NECs can be further divided into small cell and large cell types based on their histological appearance [7–10]. Immunohistochemical evidence of neuroendocrine components is demonstrated by positive staining in at least 50% of the estimated total tumour volume for synaptophysin (Syn), chromogranin A (CgA) and/or CD56 [7]. NECs may be ‘mixed-type’ if there is evidence of adenocarcinoma or squamous-cell carcinoma in addition to at least 30% of the cells staining for neuroendocrine markers.

There are limited data on the natural history and best management pathways for oesophageal NECs. Case reports on oesophageal NECs support the use of a multimodal approach utilizing both local (radiotherapy and/or surgical resection) and systemic treatment (chemotherapy) [6, 8, 9, 11, 12]. This is supported by a recent retrospective database analysis that demonstrated improved survival with the use of combination therapy [1].

We present our experience of five cases of oesophageal NEC presented to three Melbourne Hospitals between October 2012 and March 2016. Characteristics of these patients are summarized in Table 1. These cases highlight the heterogeneity of stage at presentation, the varied and often poor outcomes, and outline our treatment experience. We report these cases in line with the SCARE criteria and PROCESS criteria [13, 14].

**Table 1:** Summary of five patients with oesophageal NEC

Patient no.	Age	Gender	Stage	Histopathology	Treatment	Survival and disease status
1	67	Female	T3N1	LCNEC	Adjuvant cisplatin, 5-FU, Epirubicin. Oesophagectomy	NOD 8 years post-diagnosis
2	67	Male	T2N0	SCNEC	Oesophagectomy. Recurrence managed with palliative carboplatin and etoposide.	20 months. DOD
3	63	Male	T3N2	SCNEC	Neoadjuvant carboplatin and etoposide. Oesophagectomy. Adjuvant carboplatin and etoposide. Palliative doxorubicin, vinciristin and cyclophosphamide.	18 months. DOD
4	63	Male	TxN ≥ 1 M1	LCNEC	Palliative etoposide. Changed to FOLFORI. Palliative Stent.	12 months. DOD
5	68	Male	TxN ≥ 1 M1	Mixed-type SCNEC	Palliative carboplatin and etoposide. Palliative Stent.	5 months. DOD

NOD, no evidence of disease

DOD, dead of disease

## CASE SERIES

### Case 1

**Figure 2:**
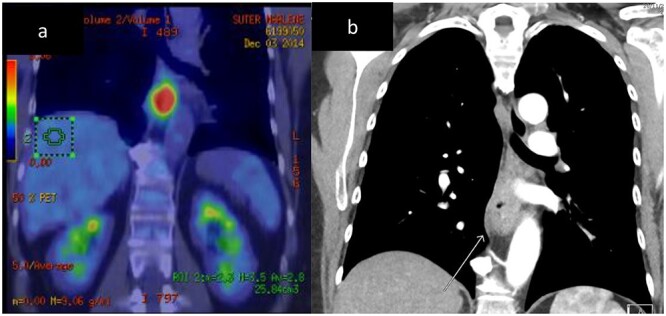
Case 1. CT and FDG-PET imaging of primary oesophageal tumour. Thickening of the oesophageal wall **(a)** and intense uptake on FDG-PET **(b)**.

A 67-year-old (yo) woman was diagnosed with 1 month of dysphagia. She had no medical history. Endoscopic examination demonstrated a circumferential tumour in the mid to distal oesophagus with biopsies confirming a large cell neuroendocrine carcinoma (LCNEC) positive for CD56, Cga and cytokeratin (CK) 5 and 6. The Ki-67 index was 70%. Computed tomography (CT) demonstrated a thickened mid-oesophagus with lymphadenopathy of one para-tracheal node, measuring 1.9 cm (Fig. 2). Fluorodeoxyglucose (FDG) positron emission tomography (PET) scan indicated intense uptake of the mid-oesophageal mass without evidence of other metastases (Fig. 2). She underwent endoscopic ultrasound (EUS) and fine need aspirate (FNA) of the para-tracheal node which confirmed metastases. She was managed with neoadjuvant cisplatin, 5-fluourouracil (5-FU) and epirubicin. After 5 months following her diagnosis, she underwent a two-stage oesophagogastrectomy (Ivor–Lewis). Histopathology revealed a LC NEC with pathological stage of T3N1. One out of nine lymph nodes demonstrated metastases. No lymphovascular or perineural invasion was seen. The patient’s post-operative course was complicated by a pleural effusion, functional decline and swallowing difficulties; however, she improved and was discharged on day 18.

Due to her complicated post-operative course, she was not fit for adjuvant chemotherapy. At 3, 9 and 10 months, post-operatively, the patient underwent endoscopic dilatation of an anastomotic stricture. The patient is currently well and undergoing routine surveillance 8 years following her diagnosis.

### Case 2

A 67yo man was diagnosed with a 3-month history of dysphagia and odynophagia. He had a medical history of 20-pack-years of smoking. Endoscopic examination demonstrated a mid-oesophageal hemi-circumferential tumour, and a biopsy diagnosed a small-cell neuroendocrine carcinoma (SC NEC), positive for CgA, Syn and CK7. The Ki-67 index was 70%. A CT and FDG PET scan did not demonstrate any metastatic disease. He underwent a three-stage oesophagectomy and had an uncomplicated recovery.

**Figure 3:**
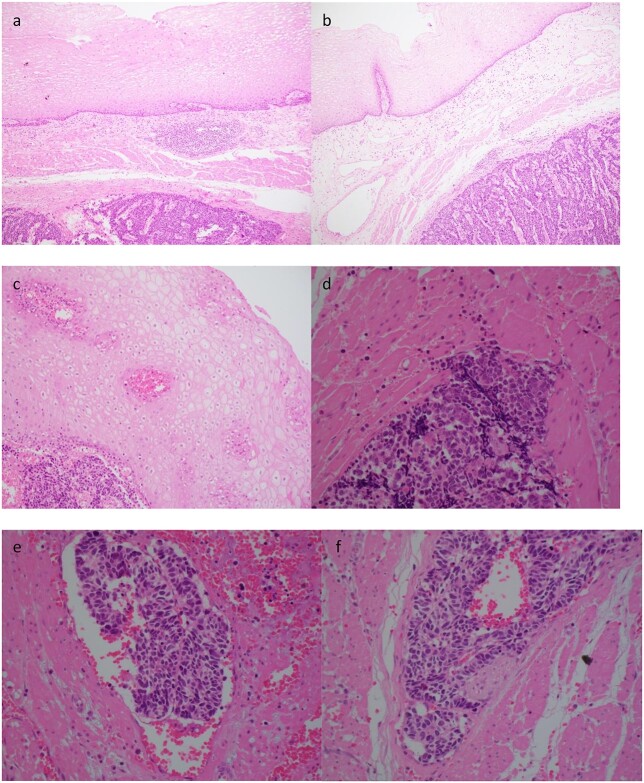
Case 2. Histopathology of the primary tumour. **(a)** and **(b)** demonstrates intact overlying oesophageal squamous mucosa, undermined by the infiltrative small cell lesion with invasion of the lamina propria **(c)**, muscularis propria **(d)**, vessels **(e)** and nerves **(f)**.

Histopathology demonstrated an SC NEC with pathological stage T2N0. Lymphovascular space invasion was present (Fig. 3). Nine lymph nodes were negative for metastases.

The patient did not undergo any adjuvant treatment; however, local and distant recurrence was detected on routine surveillance CT scan 8 months post-operatively, with a soft tissue mass adjacent to the gastric conduit and a 40-mm new liver lesion in segment VIII. Serum CgA was elevated at 30 U/L (range 1–21.8). The patient underwent six cycles of chemotherapy with carboplatin and etoposide, which were tolerated well. CT imaging initially demonstrated a response to chemotherapy; however, after four cycles, there was an increase in the tumour size. The patient then opted for a period of observation and was diagnosed 2 months later with symptoms of stridor, headaches and neurological symptoms. CT imaging demonstrated four intra-cerebral lesions consistent with metastases, increased size and invasion of the para-oesophageal mass, mediastinal lymphadenopathy and multiple new liver lesions consistent with progressive disease.

Palliative therapy was commenced, and the patient died 20 months after surgery.

### Case 3

A 63yo man was diagnosed with a history of haematemesis. He had a medical history of intermediate-risk prostate cancer, hypertension, hypertrophic cardiomyopathy, coronary artery disease, strokes and obstructive sleep apnoea. Endoscopic examination demonstrated an exophytic tumour at the gastro-oesophageal junction (GOJ) with biopsies demonstrating an SC NEC with positive staining for CD56 and CK8/18 (CgA and Syn staining not performed). CT and PET imaging indicated a GOJ primary with nodal metastases to right para-oesophageal, and left gastric nodes.

The patient underwent three cycles of chemotherapy with carboplatin and etoposide. Restaging CT demonstrated reduction in the size of the oesophageal mass and lymphadenopathy, with no evidence of new metastatic disease. An endoscopy and staging laparoscopy was performed demonstrating subtle irregularity of the mucosa at the GOJ. Biopsies demonstrated residual NEC. Diagnostic laparoscopy did not demonstrate any intra-abdominal metastases, and cytology of washings was negative.

He underwent a three-stage oesophago-gastrectomy with post-operative pathology demonstrating a SC NEC with pathological stage T3N2. Lymphovascular space invasion was present. The Ki-67 index was 80%. Out of 68 lymph nodes, 6 demonstrated metastases. There was no evidence of regression of the primary tumour or lymph nodes. His post-operative period was uncomplicated.

The patient underwent a further three cycles of carboplatin and etoposide chemotherapy starting 2 months post-operatively.

CT scan 8 months post-operatively demonstrated extensive metastatic disease in the liver, mediastinal, para-aortic and hilar lymph nodes. Palliative chemotherapy was administered containing doxorubicin, vincristine and cyclophosphamide. The patient completed eight cycles with a good clinical response. After a short break from chemotherapy, he rapidly declined and passed away 18 months following his diagnosis.

### Case 4

**Figure 4:**
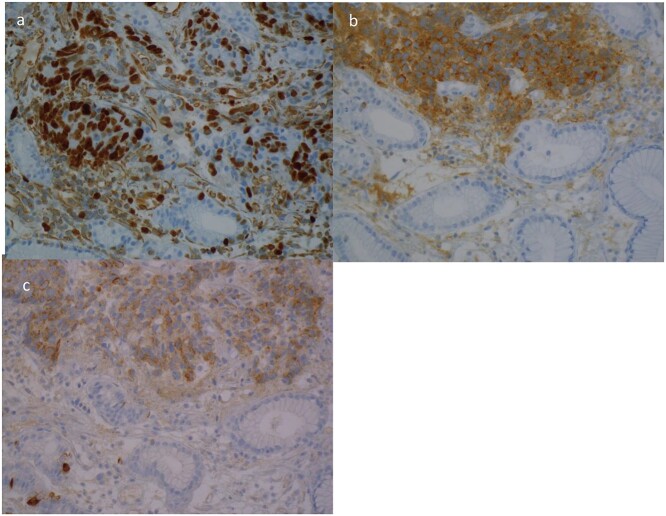
Case 4. Immunohistochemical profile of the tumour demonstrating a classical profile for neuroendocrine carcinoma. **(a)** Ki67, **(b)** synatophysin, **(c)** CgA.

A 63yo man was diagnosed in October 2012 with 3 months of progressive dysphagia and weight loss. He had a history of hypertension. Endoscopic examination demonstrated an ulcerated Siewert III GOJ tumour. Biopsy specimens demonstrated an LC NEC; positive for CgA, Syn and CK7 (Fig. 4). The Ki-67 index was >50%. CT and PET staging demonstrated a bulky GOJ primary, lymphadenopathy, and multiple pulmonary and liver metastases consistent with metastatic disease.

An oesophageal stent was placed and the patient underwent palliative chemotherapy with etoposide for 3 months, and was then changed to FOLFIRI chemotherapy. The patient passed away 12 months following the diagnosis.

### Case 5

**Figure 5:**
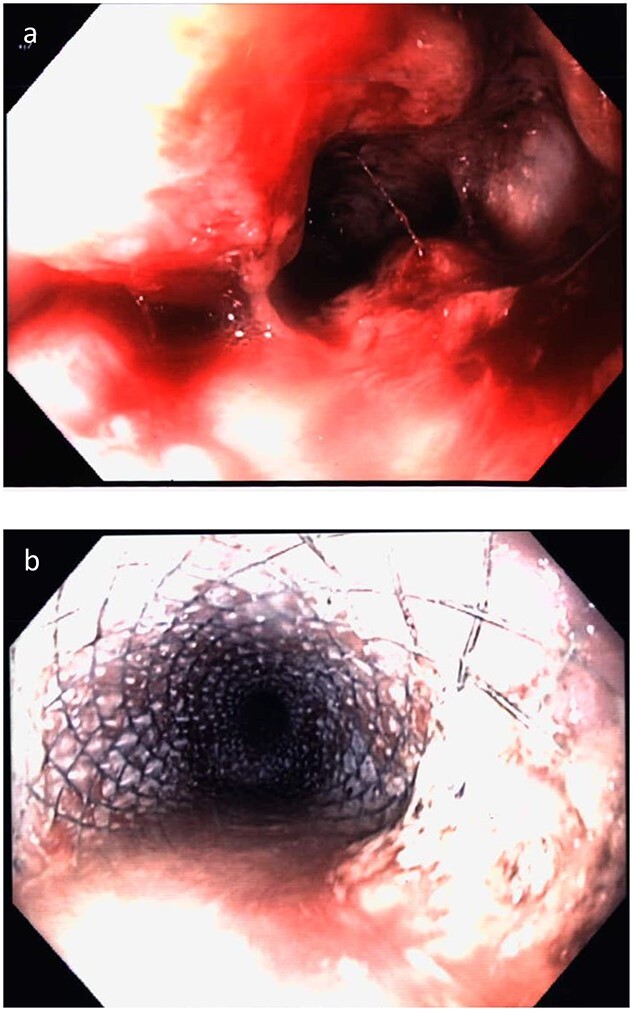
Case 5. Endoscopy demonstrating **(a)** ulcerated stenosing tumour at the distal oesophagus and **(b)** oesophageal stent *in situ*.

A 68yo man was diagnosed with a 4-week history of progressive cough, shortness of breath and dysphagia. He had a history of Wegener’s granulomatosis and diverticulosis. Endoscopic examination demonstrated a nearly-obstructing ulcerated mass in the distal oesophagus (Fig. 5). Endoscopic biopsies demonstrated a mixed-type SC NEC, with some adenocarcinoma component; positive for syn, and negative for CD56, CgA, p63, CK7 and CK20. The Ki-67 index was >80%. CT and PET staging demonstrated a distal oesophageal primary with para-oesophageal lymphadenopathy and multiple hepatic metastases. USS guided liver biopsy confirmed metastatic mixed-type SC NEC.

The patient underwent carboplatin and etoposide-based chemotherapy with re-staging CT initially demonstrating a response to treatment, but after four cycles, there was progressive disease. An oesophageal stent was inserted after the fourth cycle for symptom management (Fig. 5). Chemotherapy was ceased due to poor treatment response and patient wishes, and he passed away 5 months following the diagnosis.

## DISCUSSION

Oesophageal NENs represent 0.05–2.4% of all oesophageal neoplasms [9, 11]. They present at a mean age of 55.8–63 years, with 22–42% of patients presenting with synchronous and 4–10% with metachronous metastases [8, 15, 16]. Risk factors include smoking, alcohol consumption and increasing age [1]. Presenting symptoms include dysphagia, retrosternal pain, loss of weight or appetite, melaena, haematemesis, GORD or symptoms related to metastases [7, 8, 11, 15, 17]. Oesophageal NENs rarely present with systemic hormonal syndromes [17].

Diagnosis and staging involves a combination of endoscopy, radiology and histological studies. Endoscopy and multiple biopsies are recommended for diagnostic certainty [10]. EUS may be utilized for local staging, as well as the assessment of loco-regional nodes [8, 16]. Contrast-enhanced CT scans are used for staging and may be supplemented by magnetic resonance imaging of the liver, which has high sensitivity for liver metastases [9, 16]. Metabolic imaging with PET scans is positive in 100% of NEC, however, is less reliable in NET [6, 16, 18]. PET imaging of Somatostatin receptors such as ^111^In-Octreoscan or ^68^Ga-DOTATOC PET is more likely to be positive in NETs (79–85%); however, it is also positive in 57% of NECs [16, 18]. Serum tumour markers such as neuron-specific endolase and CgA may be positive and can be used to monitor treatment response and recurrence [10, 16]. Staging laparoscopy with peritoneal washings is a standard component of the workup for lower oesophageal adenocarcinoma and squamous cell carcinoma. The use of these modalities in oesophageal NENs is not established but was utilized by our institution for one patient.

Treatment of oesophageal NECs is not well defined due to the rarity of these tumours. There are no established treatment pathways or prospective randomized trials, with most studies limited to retrospective case series. Management of oesophageal NECs is typically multimodal, utilizing both local (radiotherapy and/or surgical resection) and systemic treatment (chemotherapy) [6, 8, 9, 11, 12].

Treatment options for patients with SC NEC without metastatic disease include surgery and adjuvant chemotherapy, or neoadjuvant chemotherapy/chemoradiotherapy and subsequent surgery [6, 11, 12]. Platinum-based chemotherapy, such as cisplatin and carboplatin, is used, often in combination with other agents including etoposide, irinotecan, doxorubicin or 5-fluorouracil [8–11, 15–17]. There is an argument for early systemic treatment, given the poor prognosis and frequent early distant recurrences, which support the theory that this is a primarily micrometastatic disease [19, 20]. Benefits of up-front chemotherapy include an opportunity to assess *in-situ* tumour biology, and to select patients that may most benefit from surgery. Meng et al. demonstrated a survival benefit of neoadjuvant chemoradiotherapy over surgery and adjuvant chemotherapy [20]. When neoadjuvant therapy is given, many patients will have disease progression or substantial tumour remaining at resection [8].

Treatment options for patients with SC NEC and evidence of metastatic disease include systemic treatments with chemotherapy and radiotherapy for local control. Most patients will progress with treatment [8, 10, 11, 15, 16]. Chemotherapeutic agents used are the same as for limited disease, with further options in case of progression including temozolomide, capecitabine, platinum-based chemotherapy or a FOLFIRI combination with temozolomide, irontecan and 5-fluorouracil [10, 16, 17].

Data on the management of LC NEC are limited and mostly extrapolated from the management of SC NEC. In Maru et al. platinum-based chemotherapy was utilized as a first approach for LC NEC where there was no evidence of metastatic disease [8]. Out of 10 patients, 8 then had surgical resection with the majority having greater than 50% residual disease in the resection specimen, and two patients progressing on treatment [8]. There are insufficient data to determine whether SC and LC NEC should be treated differently [10].

Radiotherapy is utilized in the literature for NEC in combination with surgery or chemotherapy. Limited data are available on its use; however, retrospective SEER database studies have demonstrated a survival benefit with radiotherapy, and further benefit when used as part of multimodal therapy [1, 12].

Future therapies currently being studied include immunotherapy, which has some early promising evidence of potential benefit for G3 NECs [21].

## CONCLUSION

Oesophageal NENs are rare and, compared with other oesophageal neoplasms, are associated with a very poor prognosis. There is no clear evidence-based management pathway for their treatment due to a lack of prospective research, and therefore a multimodal treatment plan needs to be individualized based on patient and tumour factors. Further research and publications on this topic should focus on collaboration to establish treatment pathways, which improve the outcomes for patients.
